# Lessons to Learn From Low-Dose Cyclosporin-A: A New Approach for Unexpected Clinical Applications

**DOI:** 10.3389/fimmu.2019.00588

**Published:** 2019-03-28

**Authors:** Camila Flores, Guillemette Fouquet, Ivan Cruz Moura, Thiago Trovati Maciel, Olivier Hermine

**Affiliations:** ^1^INSERM UMR1163 and CNRS URL 8254, Imagine Institute, Paris, France; ^2^Paris Descartes University-Sorbonne Paris Cité, Paris, France; ^3^Department of Hematology, Necker Children's Hospital, APHP, Paris, France

**Keywords:** cyclosporin-A, low-dose, oncology, lymphocytes, immunotherapy, cytokines

## Abstract

Cyclosporin-A has been known and used for a long time, since its “fast track” approval in the early 80's. This molecule has rapidly demonstrated unexpected immunosuppressive properties, transforming the history of organ transplantation. Cyclosporin's key effect relies on modulation on T-lymphocyte activity, which explains its role in the prevention of graft rejection. However, whether cyclosporin-A exerts other effects on immune system remains to be determined. Until recently, cyclosporin-A was mainly used at a high-dose, but given the drug toxicity and despite the fear of losing its immunosuppressive effects, there is nowadays a tendency to decrease its dose. The literature has been reporting data revealing a paradoxical effect of low dosage of cyclosporin-A. These low-doses appear to have immunomodulatory properties, with different effects from high-doses on CD8+ T lymphocyte activation, auto-immune diseases, graft-vs.-host disease and cancer. The aim of this review is to discuss the role of cyclosporin-A, not only as a consecrated immunosuppressive agent, but also as an immunomodulatory drug when administrated at low-dose. The use of low-dose cyclosporin-A may become a new therapeutic strategy, particularly to treat cancer.

## Introduction

Cyclosporin-A (CsA) is a fungus-derived molecule (*Tolypocladium inflatum*), discovered in 1970 by Borel and Stahelin (1) in an attempt to develop a new antifungal treatment (2, 3). In 1971, its immunosuppressive properties were identified in a screening test (1, 4) and in 1976, its chemical structure was determined (3, 5). CsA is a neutral, lipophilic, cyclic endecapeptide consisting of 11 amino acids with a molecular weight of 1202,6 Daltons (6–8).

High-dose CsA is a very potent and relatively selective inhibitor of T lymphocyte activation, with minimal effects on already activated cytotoxic/suppressor CD8 T cells, granulocytes and macrophages, which represents a significant advantage compared to some other immunosuppressive drugs (9). In addition, it has been shown that CsA has no effect on the function of phagocytic cells, does not cause bone marrow suppression (1, 7, 10, 11), and its action on immune competent lymphocytes is reversible (1).

High-dose CsA selectively blocks T-cell receptor-induced proliferation, differentiation and cytokine production (10–13), even though activated T-cells may still be able to express interleukin 2 receptor (IL-2R) and proliferate in the presence of interleukin 2 (IL-2) (14). The main target of CsA is the T-helper cell subset, however CsA also shows a weak inhibitory effect on CD4+ CD25+ regulatory T-cells and can therefore modulate the host immune tolerance (15). There is also evidence that CsA may interfere with the function of some B cell subsets, whose co-operation with T-cells is essential for their activation (16) and antigen presentation by accessory cells (2, 3, 17, 18). CsA can hamper B cells function both directly and indirectly through interaction with T cells (19).

**Figure 1:**
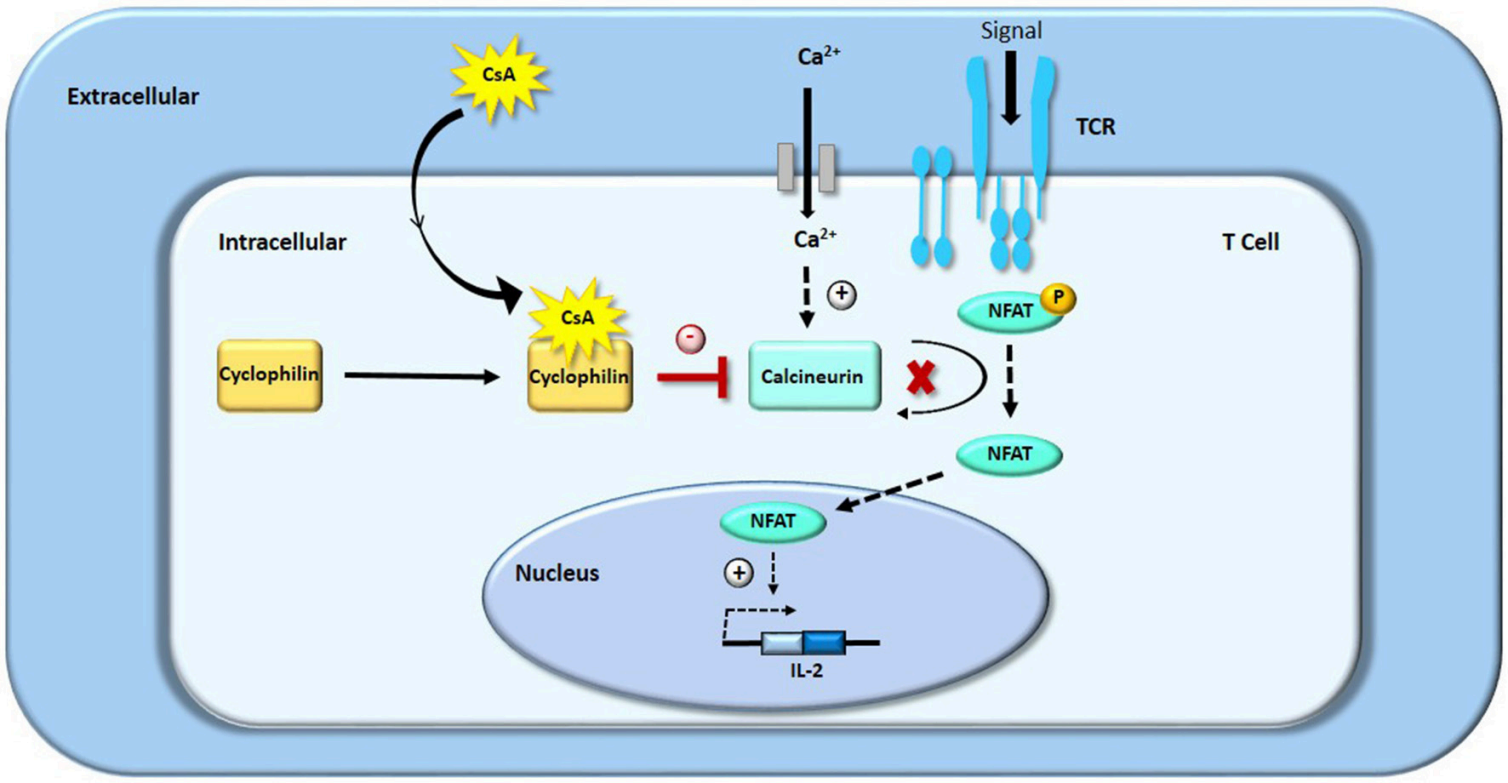
Mechanism of action of CsA: inhibition of T-cells proliferation by blocking IL-2 transcription.

CsA belongs to the family of calcineurin inhibitors (for further information on CsA mechanism of action please see Figure 1). CsA binds with great affinity to cyclophilins, and particularly to the cytosolic 17 kDa cyclophilin-A, a family of cytoplasmic proteins present in most of the T-cells (11, 20, 21). The drug-receptor complex specifically and competitively binds to calcineurin, a calcium/calmodulin dependent serine threonine protein phosphatase, provoking its inhibition (11, 22). This process restricts the dephosphorylation of a family of transcription factors, the nuclear factor of activated T-cells (NFAT) family (23). High-dose CsA prevents NFAT's translocation from the cytoplasm to the nucleus, decreasing the transcription of several immunologically important factors such as interleukins (IL-2, IL-3, IL-4), tumor necrosis factor alpha (TNF-α) and interferon-gamma (IFN-γ) (21, 22, 24, 25). For instance, it has been demonstrated that high-dose CsA—but not low dose CsA—directly inhibits the expression of NFATc1 and IL-2 in human T cells from stimulated whole blood samples, assessed by flow cytometry (23). Therefore, unlike other cytotoxic immune suppressors, CsA does not kill the immune effector cells but rather selectively inhibits their proliferative activation and expansion, mainly by interfering with IL-2 synthesis, which is essential for the activation and differentiation of T lymphocytes.

Upon administration, CsA is found widely distributed in the extravascular space, with an average apparent distribution volume of 3.5 L/kg. In the blood, the distribution is as follows: 33–47% in plasma, 4–9% in lymphocytes, 5–12% in granulocytes and 41–58% in red blood cells. In plasma, about 90% of CsA is bound to proteins, mainly lipoproteins (7).

CsA is metabolized mainly by the liver to about 15 metabolites, through the cytochrome P450 3A4 (CYP3A4) and P-glycoprotein. The main metabolic pathways involved are monohydroxylation, dihydroxylation, and N-demethylation at different positions within in the molecule (26).

The bioavailability of CsA is influenced by its intestinal absorption, which leads to inter-individual variability (27). Several factors are known to have an impact on intestinal absorption, such as: time post-graft, bile flow, dietary composition, gastrointestinal state, liver function, small bowel length and vehicle of CsA (15). The elimination occurs essentially by biliary excretion and feces (11), with only 6% of the dose excreted in the urine after oral administration (8). The terminal elimination half-life varies from 6.3 h in healthy volunteers to 20.4 h in patients with severe hepatic disease. The half-life in renal transplant patients is ~11 h, ranging from 4 to 25 h (26, 28). The time to peak blood CsA concentration ranges from 1.2 to 2 h following oral administration.

## CsA in Clinical Settings

### High-Dose CsA

Rapidly after its discovery, CsA has changed paradigms in the immunosuppression field due to its ability to prevent acute organ rejection and thus improve overall survival in kidney (29) and in liver transplant (30). The first treated patient was a 28-year-old woman who received a liver transplant in 1980 (30).

The success of the clinical use of CsA led to its rapid approval by the Food and Drug Administration (FDA) in 1983 (31) and it has fundamentally transformed the field of organ transplantation compared to other immune suppressants (32). Beside its role to prevent rejection after solid organ transplantation, CsA is also indicated for preventive or curative treatment of graft-vs.-host disease (GVHD) (33) and treatment of inflammatory disorders such as psoriasis (34), atopic dermatitis (35), nephrotic syndromes (36), or rheumatoid arthritis (37).

Adverse effects of CsA include: nephrotoxicity, neurotoxicity (convulsions, encephalopathy, anxiety, and headache), hepatotoxicity, cardiovascular toxicity (hypertension, arrhythmia), diarrhea, endocrinological, and metabolic toxicity (dyslipidemia, hypomagnesaemia, hyperkalemia, gynecomastia, and hypertrichosis). Unfortunately, these undesirable toxicities emerged simultaneously with CsA's immunosuppressive benefits. In the early era, CsA was used at high doses, up to 25 mg/kg per day following transplant (7, 29). These unacceptable toxicities then led to a necessary dose reduction.

### Low-Dose CsA

If doses above 20 mg/kg per day can be defined without doubt as high doses, the definition of “low-dose” CsA is less clear. In general, CsA is initially administrated up to 20 mg/kg per day following solid organ transplant in adults, then decreased every week until 5 to 10 mg/kg per day. In auto-immune and inflammatory diseases, CsA is rather used at doses around 4 to 6 mg/kg per day. After allogeneic hematopoietic stem cell transplantation (HSCT), CsA is often prescribed at 1 mg/kg per day and adjusted to provide therapeutic blood levels from 150 to 400 ng/mL (38), even though the target concentration can vary depending on clinical protocols, type of allograft, risk of rejection, concomitant immunosuppressive medications, and toxicity (32, 38). Doses below 3 mg/kg per day are generally considered as low doses, and doses above 4 mg/kg can already be considered as high doses depending on studies.

The first attempts of lowering CsA doses aimed at decreasing its side effects while maintaining optimal efficacy. The nephrotoxicity of CsA correlates with duration of treatment and dose, and is reversible after dose reduction. The fear of losing the immunosuppressive effect of CsA due to dose reduction led to the development of protocols including antilymphocytic antibodies in the early post-transplant period (39), also allowing to avoid early CsA exposure before full recovery of the allograft function. Subsequently, decreasing the dose of CsA demonstrated improved outcomes in organ transplant (4, 40).

As detailed below, several reports revealed a paradoxical effect of low dosage CsA, such as immunomodulatory properties (14, 41), anti-GVHD (42), and anti-cancer effects (43).

## Clinical Applications for Low-Dose CsA

### Immunomodulatory Properties

If high-dose CsA inhibits T-cells activation, evidence shows that low-dose CsA can induce pro-inflammatory cytokines, as well as autoimmunity and immune hyperreactivity (14). The suppressive activity of CsA on T lymphocytes was first challenged by Bretscher et al. (44), who found that low-dose CsA can activate the cell-mediated immune response (Table 1). It has also been suggested that low-dose CsA inhibits regulatory T-cells activity *in vitro* whereas this effect is weak with higher doses (45). Low-dose CsA may therefore be considered to stimulate immune response in specific conditions (41).

**Table 1:** Characterization of cyclosporin-A (CsA) dose effects.

	**High-dose CsA**	**Low-dose CsA**
Dose per day	≥4–5 mg/kg	≤ 3 mg/kg
Target concentration	>150 ng/ml	<150 ng/ml
T-cell proliferation	Decreased/abolished	Maintained
Cytokine production	Decreased/abolished	Maintained
Increased auto-immunity	No	Yes
Increased anti-cancer immunity	No	Yes

In mice models, it has been shown that low-dose CsA induces pro-inflammatory cytokines such as IL-12, IFN-γ and TNF-α (46). Another study demonstrated that mice treated with low-dose CsA (5 × 10^−55^ mg/kg per day) presented an accelerated allograft skin rejection, a decreased CD4^+^ CD25^hi^ FoxP3^+^ regulatory T-cell subpopulation, and an activation of innate immunity, when compared to animals receiving high-dose CsA (15 mg/kg per day) or placebo (46).

Under some circumstances, CsA can paradoxically augment delayed-type hypersensitivity responses, aggravate autoimmune diseases and induce specific forms of autoimmunity (14). The authors reported that CsA can aggravate and/or induce relapse in several autoimmune diseases including collagen-induced arthritis, encephalomyelitis and autoimmune thyroiditis, suggesting that CsA may enhance immune responses by inactivating suppressor cells, altering Th1/Th2 antagonism or promoting T cell activation through a CsA-resistant IL2-independent T cell activation (14).

In a study on experimental allergic encephalomyelitis (EAE) in rats, low-dose CsA (3 mg/kg per day) had a minor protective effect during the acute disease state (day 10 to 17 post-immunization) (47). However, while EAE control rats recovered from the disease, rats treated with low-dose CsA presented a severe disease relapse 20–30 days post immunization, whereas high-dose CsA (20 mg/kg per day) completely protected rats from EAE. This relapse was associated to increased numbers of cells spontaneously producing IFN-γ in the central nervous system and regional lymph nodes. The authors also showed an increase of anti-myelin and anti-MBP (myelin basic protein) secreting cells, as well as primed T cells that produced IFN-γ in response to myelin antigens. They suggested that low-dose CsA could interfere with systemic down-regulatory mechanisms acting on both T-cell and B-cell myelin-directed autoimmunity.

### GVHD

Immunosuppression is crucial in solid organ transplant to prevent rejection, but also in allogeneic HSCT to prevent GVHD. CsA associated with a short course of methotrexate has been the most common immunosuppressive regimen in allogeneic HSCT since CsA's approval in 1983 (5, 48). CsA was initially used at doses up to 10 to 20 mg/kg per day, and subsequently lowered to 3 to 5 mg/kg per day because of unacceptable toxicity.

It has been shown that high-dose CsA efficiently reduces the incidence of severe GVHD, but also decreases the graft-vs.-leukemia (GVL) effect, increasing the rate of leukemic relapses (49). In contrast, Olsson et al. (42) demonstrated that low-dose CsA (1 mg/kg I.V per day) improved survival in leukemic recipients of HLA-identical sibling transplants in comparison with high-dose CsA (5 to 7.5 mg/kg I.V per day). This retrospective study reported that, compared to patients on high-dose CsA, patients on low-dose CsA had an increased probability of developing acute GVHD grades I-II (70 vs. 53%, *p* < 0.01), and chronic GVHD (58 vs. 25%, *p* < 0.01), whereas the incidences of acute GVHD grades III–IV (9 vs. 5%, *p* = 0.62) and non-relapse mortality (20 vs. 22%, *p* = 0.58) were similar. This was associated with a decreased probability of relapse (31 vs. 54% *P* < 0.01) and an improved relapse-free survival (56 vs. 38%, *p* < 0.04) and overall survival (61 vs. 40%, *p* < 0.04) under low-dose CsA regimen. In multivariate analyses, low-dose CsA remained strongly associated with chronic GVHD (hazard ratio 2.56, *p* < 0.01), decreased risk of relapse (hazard ratio 0.46, *p* < 0.01) and increased probability of survival (hazard ratio 1.84, *p* < 0.01).

These results suggest that low-dose CsA used for GVHD prophylaxis in allogeneic HSCT could improve survival by improving GVL effect without increasing severe acute GVHD—keeping in mind that this is a retrospective study, restricted to HLA-identical sibling transplants recipients treated for leukemia.

To note, it has also been suggested that selective NFAT targeting in T cells could improve GVHD while maintaining the beneficial GVL effect (50).

### Cancer

Anti-cancer therapy is perhaps the most significant application for low-dose CsA and its putative role in immune system activation. In a phase I/II trial on 44 patients with advanced non-small cell lung carcinoma (NSCLC), low-dose CsA (1-2 mg/kg per day) was compared to high-dose CsA (3-6 mg/kg per day) in association with Etoposide and Cisplatine (43). In this small series, the authors reported a significant increase in survival of patients treated with low-dose CsA, with a 2-year survival of 25% compared to 4% with high-dose CsA. The Kaplan-Meier survival curves were significantly different for these two groups by the log-rank test (*p* = 0.047). Although no conclusions should be drawn from this small study, this suggests that low-dose CsA could, in some situations, be a therapeutic approach in cancer.

CsA is known to be a specific inhibitor of the nuclear factor of activated T-cells (NFAT) pathway (Figure 1), and emerging evidence suggests that NFAT signaling plays an important role in tumorigenesis and tumor growth. The overexpression of several isoforms of NFAT has been detected in pancreatic, pulmonary or hepatic carcinomas, as compared with their corresponding normal tissues (51–53). The isoform NFATc1 seems to act as an oncogene as a constitutively active form of NFATc1 is able to induce neoplastic transformation of fibroblast cells, whereas the isoform NFATc2 rather seems to act as a tumor suppressor (54). NFATc1 has been shown to promote tumor progression in pancreatic cancer (51), melanoma (55), and breast cancer (56), and to be implicated in lymphangiogenesis (57). Some studies have demonstrated an antitumor effect of various doses of cyclosporin A in bladder (58) and prostate cancer (59).

At high doses, it has been shown that CsA is able to reduce the growth of cancer cells *in vitro*, by inducing cycle cell arrest, apoptosis or necroptosis in colon cancer (60, 61), gastric cancer (62) or squamous cell carcinoma (63). CsA's impact in these studies however seems to be mainly tumoristatic, and is not observed for all cancer cells: for instance, breast cancer cells are refractory to CsA (60). To our knowledge, no published study has demonstrated a direct effect of low-dose CsA on cancer cells.

In hematology, low-dose CsA has also been used in combination with Cytarabine and Daunorubicine in patients with acute myeloid leukemia (64). The complete hematological remission rate after the first cycle of induction was higher in the CsA group (63.6 vs. 30%; *p* = 0.09). However, relapse rate and mortality were higher in the CsA group, resulting in the lack of significant improvement in outcome. Despite these disappointing results, the increase of response rate can suggest an anti-leukemic effect of low-dose CsA.

Considering the action of CsA on T-cells, it was suggested that CsA could be potentially effective in the treatment of T-cell neoplasms. A study was performed in 16 patients with refractory T-cell lymphoma (peripheral T-cell lymphoma or cutaneous T-cell lymphoma) using high-dose CsA (15 mg/kg per day) (65). Most patients in this trial did not respond to high-dose CsA, suggesting either that these malignancies are IL-2 independent or that CsA could not reach its intracellular target. Only two patients responded to high-dose CsA, but rapidly relapsed after a temporary halt in therapy, arguing for a cytostatic or anti-inflammatory effect rather than a cytotoxic effect of CsA. However, this study has limitations regarding our subject of interest since it assessed only high-dose CsA and lack of efficacy of CsA monotherapy in these high-risk diseases could be expected. To our knowledge, no study has been conducted with low-dose CsA on hematologic neoplasms, therefore, CsA's role on anti-tumor immunity in this setting remains unknown.

More recently, a case report described a remarkable efficacy of CsA in a patient with advanced thymoma (66). CsA was used at 5 mg/kg per day and was then adjusted to maintain serum levels of 100–150 ng/ml, corresponding to a low-dose CsA regimen. The patient, initially diagnosed with a microinvasive thymoma and subjected to thymectomy, relapsed 10 years later with the same histology. After being treated with methylprednisolone pulses followed by a complete resection of the recurrent thymoma, the patient relapsed with disseminated recurrent thymoma and pure red blood cell aplasia, for which she received CsA. Interestingly, the patient was cured for red blood cell aplasia and all thymoma lesions disappeared without any additional therapy.

The potential therapeutic window of CsA appears to be relatively narrow in the context of cancer, particularly because of its controversial effect on regulatory T cells (T-regs). In some studies, low-dose CsA has been shown to inhibit T-regs activity *in vitro* whereas this effect was weak with higher doses (45). However, in patients with atopic dermatitis, low-dose CsA increased (67), while high-dose CsA reduced (68), the T-reg population. In mice, treatment with low-dose CsA was shown to decrease the T-reg population and accelerate graft rejection (46), while low-dose CsA induced tolerance in a model of kidney graft with strong histoincompatibility in rats (69). In another study in mice, high-dose CsA inhibited T-regs and impaired their immunosuppressive function (70). Since T-regs are usually considered to promote tumors, low-dose CsA could be detrimental in some cancer settings. However, T-regs are a heterogeneous subset of immunosuppressive T cells, which do not always favor tumor progression and can be beneficial to the patient (71).

In summary, even though clinical data are scarce, low-dose CsA could represent an effective, safe, low-cost therapy for several kinds of cancer. However, the impact of low-dose CsA on tumors requires further studies to increase our understanding of its immunological effects.

## Conclusion and Perspectives

CsA is a molecule known and used for almost 40 years for its immunosuppressive properties. Over time, it has been revealed that CsA is able to activate or inhibit the immune system, in a dose dependent manner. Thus, one could suggest several possible uses of CsA, adapting the dose to the desired impact. Low-dose CsA seems to have a negative effect on situations like hyper-reactivity and autoimmune diseases. However, in cases such as allogeneic HSCT and GVHD prophylaxis, low-dose CsA has proven its efficacy. There are significant arguments to support low-dose of CsA as a promising strategy in the arsenal against cancer, whether as monotherapy or in combination with drugs. Further investigations on its therapeutic efficacy and prognosis impact are yet needed. We are currently observing an increased use of immunotherapies such as anti-checkpoint inhibitors or CAR-T cells. CsA combination may modulate immunotherapies either at high-dose, decreasing their toxicities, or at low-dose to increase their efficacy. Low-dose CsA may give rise to major contributions to medicine and notably to the field of oncoimmunology.

## Author Contributions

All authors listed have made a substantial, direct and intellectual contribution to the work, and approved it for publication.

### Conflict of Interest Statement

The authors declare that the research was conducted in the absence of any commercial or financial relationships that could be construed as a potential conflict of interest.

## Abbreviations

CsA: cyclosporin-A
EAE: experimental allergic encephalomyelitis
GVHD: graft-vs.-host disease
GVL: graft-vs.-leukemia
HLA: human leukocyte antigen
HSCT: hematopoietic stem cell transplantation
IL-2: interleukin 2
IL-2R: interleukin 2 receptor
IFN-γ: interferon-gamma
NFAT: nuclear factor of activated T-cells
TNF-α: tumor necrosis factor alpha.
